# Run‐in periods and clinical outcomes of antipsychotics in dementia: A meta‐epidemiological study of placebo‐controlled trials

**DOI:** 10.1002/pds.4903

**Published:** 2019-11-15

**Authors:** Tessa A. Hulshof, Sytse U. Zuidema, Christine C. Gispen‐de Wied, Hendrika J. Luijendijk

**Affiliations:** ^1^ Department of General Practice and Elderly Care Medicine University Medical Center Groningen Groningen The Netherlands; ^2^ Dutch Medicines Evaluation Board Utrecht The Netherlands

**Keywords:** antipsychotics, dementia, efficacy, meta‐analysis, pharmacoepidemiology, run‐in, side effects

## Abstract

**Purpose:**

Run‐in periods are used to identify placebo‐responders and washout. Our aim was to assess the association of run‐in periods with clinical outcomes of antipsychotics in dementia.

**Methods:**

We searched randomized placebo‐controlled trials of conventional and atypical antipsychotics for neuropsychiatric symptoms (NPS) in dementia in electronic sources and references of selected articles. We extracted (a) the presence of a run‐in period, use of placebo/investigated drug during run‐in (versus washout only), and run‐in duration (1 week or more) and (b) the reduction in NPS, number of participants with somnolence, extrapyramidal symptoms (EPS), and deaths per treatment group. We pooled clinical outcomes comparing antipsychotic and placebo groups in trials with and without run‐in.

**Results:**

We identified 35 trials. Twenty‐nine trials used run‐in. The pooled standardized mean difference in the reduction of NPS was −0.170 (95% CI, −0.227 to −0.112) in trials with run‐in and −0.142 (95% CI, −0.331 to 0.047) in trials without run‐in. The pooled odds ratio for somnolence was 2.8 (95% CI, 2.3‐3.5) in trials with run‐in and 3.5 (95% CI, 1.2‐10.7) in trials without run‐in; for EPS, these ORs were 1.8 (95% CI, 1.4‐2.2) and 2.0 (95% CI, 1.3‐3.1) respectively, and for mortality 1.4 (95% CI, 1.0‐2.0) and 1.6 (95% CI, 0.7‐3.4). The use of placebo/investigated drug during run‐in and run‐in duration did not affect the estimates in a consistent way.

**Conclusions:**

The use of run‐in in trials might have led to overestimated efficacy and especially underestimated risks of side effects of antipsychotics compared with placebo for NPS in dementia.

**Key Points**

- Run‐in periods have been used in the majority of trials that tested conventional and atypical antipsychotics in dementia.
- The use of run‐in periods increased the estimated efficacy of antipsychotics in dementia.
- The use of run‐in periods decreased the risk of somnolence, extrapyramidal symptoms, and mortality of antipsychotics in dementia.
- The use of run‐in periods did not affect drop‐out substantially.
- The effect of run‐in on clinical outcomes of trials needs to be addressed as part of reviews.

## 1 INTRODUCTION

Results of randomized controlled trials are important for regulatory and clinical decisions. Researchers have therefore sought to optimize treatment effects and identify patients that will benefit most from treatment. One way of enhancing trial design is using a run‐in period between screening for eligibility and before randomization.^1^, ^2^ During this period of usually 1 to 2 weeks, drugs that the eligible patients already used are washed out. In some trials, the drugs are replaced by placebo to blind the participants for the change in treatment. Drug‐naïve patients can also be given placebo, or the active drug of interest. Patients with high placebo response, poor compliance, low treatment response, or intolerance for the drug can thus be identified.^3^, ^4^ At the end of the run‐in phase, the researchers select the participants that are definitively included in the study. It is assumed that a run‐in period will decrease placebo response and dropout during the trial and consequently increase the effect size and the power of a trial.^5^, ^6^

A small number of reviews have studied the effect that a run‐in period can have on trial outcomes of psychopharmacological drugs. Antidepressants in children were 15% more effective in trials with a run‐in period than in trials without a run‐in period and above the threshold for a small effect size (standardized mean difference 0.26 vs 0.17, respectively; cut‐off for small effect is 0.20).^7^ Another meta‐analysis of antidepressant trials in depressed outpatients showed that a placebo run‐in period was associated with higher efficacy and more power.^8^ On the other hand, run‐in periods were not associated with greater efficacy in trials of antidepressants for major depression, benzodiazepines for anxiety, and naltrexon for alcohol addiction.^9^, ^10^, ^11^, ^12^, ^13^

To our knowledge, the effect of a run‐in period on efficacy and side effects of antipsychotics has not been investigated before. This is notable, because high placebo response rates, high dropout rates, and decreasing effectiveness over the years are the major problems in antipsychotic trials.^14^ An association between use of a run‐in period and drug safety is not unlikely, because run‐in periods can lead to exclusion of persons not tolerating the drugs and of noncompliant subjects.^15^ Moreover, atypical antipsychotics have been marketed with the claim of a more favorable side effects profile compared with conventional antipsychotics, ie, lower rates of somnolence and extrapyramidal symptoms (EPS).^16^

Antipsychotics are often prescribed for neuropsychiatric symptoms in dementia. Trials that tested the efficacy of antipsychotics for this indication commonly used run‐in periods. The aim of this study was to assess the association of run‐in periods in trials of conventional and atypical antipsychotics in dementia with clinical outcomes and also dropout.

## 2 METHODS

We performed a meta‐epidemiological study. We wrote a research proposal for the sponsor in advance, and it can be requested from the corresponding author.

### 2.1 Search strategy

Four sources were used to identify trials. Two reviewers (T.A.H. and H.J.L.) first searched the electronic databases Cinahl, Embase, Pubmed, and Cochrane library with the strings “generic name atypical/conventional antipsychotic” AND trial AND dementia (see the Appendix S1). We composed a list of all conventional and atypical antipsychotics from the websites of the World Health Organization, Food and Drug Administration, and Wikipedia to enable this search.^17^, ^18^, ^19^ Secondly, we hand‐searched the references of published systematic reviews, which were identified with the same electronic databases. Titles and abstracts of potentially eligible studies were retrieved in Pubmed. Thirdly, we sought RCTs in trial registration websites with the same keywords where possible. Finally, we searched the databases of the Dutch Medicines Evaluation Board and the FDA for unpublished trials of atypical antipsychotics.

### 2.2 Study selection

Randomized placebo‐controlled trials that tested the efficacy of orally administered conventional or atypical antipsychotics for neuropsychiatric symptoms in dementia were included. If studies seemed potentially eligible given title and abstract, full articles were retrieved as well as online protocols of unpublished studies. Two reviewers (T.A.H. and H.J.L.) reviewed these articles for definitive eligibility. Studies with no information on the use of a run‐in period and with multiple drugs in one intervention arm were excluded. There were no restrictions with respect to publication date, language, flexible or fixed dosing of the active treatment, and duration of the study. The search was last rerun in June 2019.

### 2.3 Data extraction

Two reviewers (T.A.H. and H.J.L.) independently extracted data from the included trials. First, we extracted general study characteristics: publication year, type of antipsychotic groups, setting, type of neuropsychiatric symptoms for which the antipsychotics were tested, and total number of randomized patients. Next, we extracted characteristics of the run‐in period: presence/absence, replacement drug (no drug, placebo, or active treatment), and duration, as well as the percentage of patients excluded at the end of the run‐in phase.

We then extracted the clinical outcomes for the drug and placebo groups. Efficacy of antipsychotics in dementia can be measured with a generic instrument that covers various neuropsychiatric symptoms (eg, NPI and BEHAVE‐AD) or an instrument for specific symptoms such as agitation (eg, CMAI). We used the results measured with the instrument that matched the symptoms at enrollment, eg, if patients had to have agitation to enter a trial, we used the result reported with an agitation scale. We extracted the mean change from baseline to end point. When multiple dosages or multiple drug groups were included in a trial, an average change was calculated. We also extracted the standard deviation (SD) of the difference between the groups in mean change. If the SD was not reported, it was calculated with the *P* value, range, or confidence interval reported for the difference in mean change. Otherwise, the SD was imputed with the average of the reported SD of all trials with the same indication and instrument. For two trials that did not report the data we needed, we obtained the IPD and calculated the mean changes and SDs.^20^, ^21^ In addition, we abstracted the number of patients with somnolence (sedation and drowsiness), and with EPS, and the number that died.

Finally, we extracted the total number of patients that dropped out (total drop‐out) and the drop‐out in the groups (selective drop‐out). Run‐in is often used to decrease drop‐out and enhance power. Drop‐out is also considered to represent the balance between efficacy and side effects.^20^

The published main results' article of a trial was our primary source of information. When the article did not report the data that we needed, secondary publications, trial reports, and meta‐analysespublished online by industry were our secondary source. We contacted the authors of eligible trials to provide missing data, or individual patient data, and received such data of four studies.^20^, ^21^, ^22^, ^23^ The reviewers discussed differences in the extracted data until consensus was reached.

### 2.4 Data analyses

First, we assessed the relationship between the presence of a run‐in period with the four clinical outcomes and selective dropout. We performed meta‐analyses to pool efficacy, risk of somnolence, EPS, and mortality of the antipsychotic versus placebo groups in trials with and without run‐in periods. For efficacy in terms of reduction in NPS, we calculated standardized mean differences (SMDs) to take into account the use of different instruments in the trials. SMDs were calculated with a 95% confidence interval (CI). For risk of somnolence, EPS, mortality, and selective dropout, we calculated odds ratios (ORs) with 95% CIs. Heterogeneity, presented as *I* ^2^, was calculated for all meta‐analyses. A fixed‐effects model was applied when *I* ^2^ was below 40%, otherwise a random‐effects model.^24^

We then investigated the relationship of the characteristics of the run‐in periods with the clinical outcomes and selective dropout. The outcomes of trials that had run‐in periods with and without a placebo were pooled (we did not find trials with active drugs in the run‐in period). We also pooled outcomes of trials with a run‐in period up to 1 week versus those with a longer run‐in period (8 to 14 days). Finally, we tested the association between run‐in characteristics and total drop‐out with meta‐regression.

We ran the above analyses for all antipsychotics combined first and then for the conventional antipsychotics and atypical antipsychotics separately. We wanted to perform an a priori sensitivity‐analysis for atypical antipsychotics without quetiapine, because its efficacy and side effect profile are considered to differ. A post hoc analysis was run in which trials that had tested both an atypical drug and haloperidol were excluded from the analysis. All analyses were performed with STATA statistical software version 15.0.^25^

## 3 RESULTS

**Figure 1:**
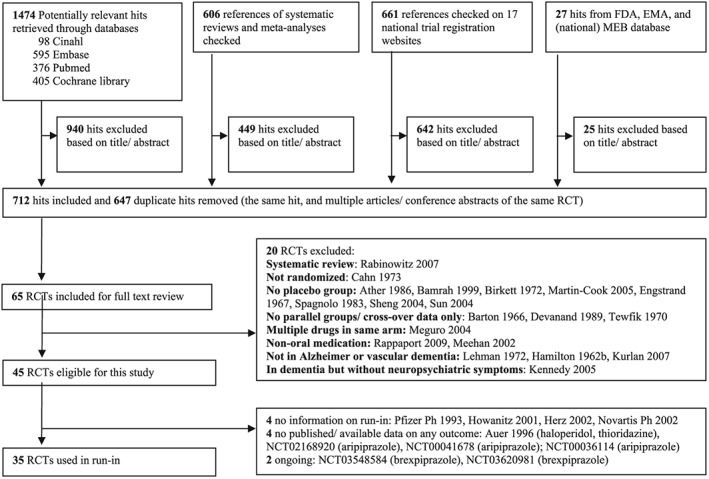
Flow diagram of literature search and study selection

Our search yielded 2768 potentially relevant RCTs (Figure 1). We obtained the reports of 65 RCTs for full text review. We identified 45 eligible RCTs, but four did not report whether a run‐in period was used, four did not report any of the outcomes of interest, and two were ongoing. We used the other 35 studies in the current study.^20^, ^21^, ^22^, ^23^, ^26^, ^27^, ^28^, ^29^, ^30^, ^31^, ^32^, ^33^, ^34^, ^35^, ^36^, ^37^, ^38^, ^39^, ^40^, ^41^, ^42^, ^43^, ^44^, ^45^, ^46^, ^47^, ^48^, ^49^, ^50^, ^51^, ^52^, ^53^, ^54^, ^55^, ^56^

Table 1 presents the general study characteristics. Twenty‐nine of the 35 studies had a run‐in period: nine of 11 conventional antipsychotic trials, 18 of 21 atypical antipsychotic trials, and two of three trials with both antipsychotics. Fourteen studies used placebo during the run‐in period, two trials the investigated drug, and 19 studies no placebo or active treatment (washout only). The duration of the run‐in periods varied between 2 days and 6 weeks. The percentage of patients excluded at the end of the run‐in period varied from 0% to 29%. In five of seven relatively old conventional antipsychotic trials, the percentage was 0%, while in the atypical trials, it was at least 7%.

**Table 1:** Randomized placebo‐controlled trials of antipsychotics in dementia with neuropsychiatric symptoms related to dementia

Author, Year	Antipsychotic	Setting	Neuropsychiatric Symptom	Run‐in Period (Duration in Weeks)	Patients Excluded After Run‐in, n (%)	Randomized Patients, n
Conventional antipsychotic trials (11)						
Hamilton, 1962	Trifluoperazine	Hospital	Diverse	No	NA	27
Sugerman, 1964	Haloperidol	Hospital	Diverse	No	NA	18
Rada, 1976	Thiothixene	Hospital	Diverse	Yes, with placebo (1)	0 (0)	42
Barnes, 1982	Thioridazine Loxapine	Nursing home	Diverse	Yes, with placebo (2)	7 (11.7)	53
Petrie, 1982	Haloperidol Loxapine	Hospital	Diverse	Yes, with placebo (2)	0 (0)	61
Stotsky, 1984	Thioridazine	Nursing home and hospital	Diverse	Yes, washout only (2)	NR	358
Finkel, 1995	Thiothixene	Nursing home	Agitation	Yes, washout only (1)	0 (0)	33
Auchus, 1997	Haloperidol	Outpatients	Agitation	Yes, washout only (2)	0 (0)	12
Devanand, 1998	Haloperidol	Outpatients	Diverse	Yes, with placebo (1)	5 (7.0)	66
Teri, 2000	Haloperidol	Hospital	Agitation	Yes, washout only (2)	0 (0)	70
Pollock, 2002	Perphenazine	Nursing home	Diverse	Yes, washout only (<1)	NR	54
Atypical antipsychotic trials (21)					
Satterlee, 1995	Olanzapine	Nursing home	Psychosis^a^	Yes, washout only	51 (17.7)	238
Janssen Ph, 1997	Risperidone	NR	Diverse	Yes, with placebo (1)	NR	39
Katz, 1999	Risperidone	Nursing home	Diverse	Yes, with placebo (1)	104 (14.3)	625
Street, 2000	Olanzapine	Nursing home	Diverse	Yes, with placebo (2)	82 (28.5)	206
Brodaty, 2003	Risperidone	Nursing home	Aggression	Yes, with placebo (1)	39 (10.2)	345
Janssen Ph, 2003	Risperidone	Nursing home	Psychosis	Yes, with placebo (1)	NR	18
De Deyn, 2004	Olanzapine	Nursing home	Psychosis	Yes, with placebo (2)	NR	652
Ballard, 2005	Quetiapine	Nursing home	Agitation	No	NA	62
De Deyn, 2005	Aripiprazole	Outpatients	Psychosis	Yes, washout only (1)	NR	208
Deberdt, 2005	Risperidone Olanzapine	Nursing home and outpatients	Psychosis	Yes, with placebo (2)	NR	494
Janssen Ph, 2005	Risperidone	NR	Psychosis	Yes, with risperidone (1)	NR	33
Mintzer, 2006	Risperidone	Nursing home	Psychosis	Yes, with placebo (1)	87 (15.5)	473
Schneider, 2006	Risperidone Olanzapine Quetiapine	Outpatients	Diverse	No	NA	421
Mintzer, 2007	Aripiprazole	Nursing home	Psychosis	Yes, washout only (1)	NR	487
Zhong, 2007	Quetiapine	Nursing home	Agitation	No	NA	333
Paleacu, 2008	Quetiapine	Not reported	Diverse	Yes, with quetiapine (2)	NR	40
Streim, 2008	Aripiprazole	Nursing home	Psychosis	Yes, washout only (1)	NR	256
Otsuka Ph, 2017a	Brexpiprazole	Nursing home	Agitation	Yes, washout only (6)	NR	413
Otsuka Ph, 2017b	Brexpiprazole	Nursing home and outpatients	Agitation	Yes, washout only (6)	NR	270
ACADIA, 2018	Pimavanserin	Nursing home and outpatients	Agitation	Yes, washout only (4)	NR	111
Ballard, 2018	Pimavanserin	Nursing home	Psychosis	Yes, washout only (3)	25 (12.1)	181
Trials with conventional and atypical antipsychotic drug group (3)					
Allain, 2000	Tiapride Haloperidol	Nursing home and hospital	Agitation	No	NA	306
De Deyn, 1999	Risperidone Haloperidol	Nursing home	Diverse	Yes, with placebo (1)	27 (7.3)	344
Tariot, 2006	Quetiapine Haloperidol	Nursing home	Psychosis	Yes, washout only (1)^b^	123 (24.6)	284

Abbreviations: NA not applicable; NR, not reported; Ph, pharmaceuticals.

a Reduction measured with generic instrument (in all other studies indication and outcome scale were congruent).

b At least 2 days.

### 3.1 Run‐in and clinical outcome

The analysis of efficacy encompassed 29 of 35 studies (Table 2). The reduction in NPS in the drug versus placebo groups was somewhat higher in trials with a run‐in period (SMD −0.170; 95% CI, −0.227 to −0.112) than in trials without a run‐in period (SMD −0.142; 95% CI, −0.331 to 0.047). Efficacy was somewhat higher when placebo or active drug was used (SMD −190; 95% CI, −0.267 to −0.112), and when run‐in lasted 1 week at most (SMD −0.214; 95% CI, −0.289 to −0.138).

**Table 2:** Use of run‐in periods and clinical outcomes of antipsychotics versus placebo in randomized trials

	Efficacy		Somnolence		EPS		Mortality	
	SMD (95% CI)	N	OR (95% CI)	N	OR (95% CI)	N	OR (95% CI)	N
Conventional and atypical antipsychotics								
No run‐in	−0.142 (−0.331 to 0.047)^a^	4	3.5 (1.2‐10.7)^a^	4	2.0 (1.3‐3.1)	5	1.6 (0.7‐3.4)	6
With run‐in	−0.170 (−0.227 to −0.112)	25	2.8 (2.3‐3.5)	16	1.8 (1.4‐2.2)	14	1.4 (1.0‐2.0)	28
‐washout only	−0.146 (−0.267 to −0.024)^a^	12	2.6 (1.4‐4.9)^a^	8	1.7 (1.1‐2.7)	5	1.2 (0.7‐2.1)	14
‐with placebo/drug	−0.190 (−0.267 to −0.112)	13	2.7 (2.1‐3.6)	8	1.8 (1.4‐2.4)	9	1.5 (1.0‐2.4)	14
‐duration ≤ 1 week	−0.214 (−0.289 to −0.138)	13	3.0 (2.1‐4.5)^a^	9	1.8 (1.4‐2.4)	10	1.4 (0.9‐2.0)	14
‐duration > 1 week	−0.108 (−0.197 to −0.019)	12	2.5 (1.7‐3.9)	7	1.8 (0.8‐4.1)^a^	4	1.5 (0.7‐3.0)	13
Conventional antipsychotics								
No run‐in	−0.389 (−0.669 to −0.110)	1	4.3 (0.2‐110.2)^a^	2	2.7 (1.4‐5.1)	2	1.4 (0.3‐6.9)	3
With run‐in	−0.345 (−0.492 to −0.199)	9	5.4 (3.2‐9.3)	4	3.0 (1.9‐4.8)	4	1.2 (0.6‐2.3)	11
Atypical antipsychotics								
No run‐in	−0.133 (−0.266 to 0.001)	4	2.8 (0.9‐8.3)^a^	3	1.6 (0.7‐3.6)^a^	4	1.6 (0.7‐3.8)	4
With run‐in	−0.141 (−0.202 to −0.081)	18	2.6 (2.1‐3.3)	14	1.6 (1.2‐2.0)	12	1.4 (1.0‐2.1)	19

Abbreviations: EPS, extrapyramidal symptoms; OR, odds ratio; SMD, standardized mean difference.

a A random effect model was used.

The number of participants with somnolence during the study period was reported in 20 of 35 studies. The pooled risk for somnolence was lower when a run‐in period was present (OR 2.8; 95% CI, 2.3‐3.5) versus when it was absent (OR 3.5; 95% CI, 1.2‐10.7). The use of placebo or active drug did not seem to affect the risk of somnolence further. The risk was lower for trials with a run‐in period of 1 week at most (OR 2.5; 95% CI, 1.7‐3.9).

Nineteen of 35 studies reported the number of participants with EPS in the treatment groups. The risk of EPS in trials with a run‐in period was slightly lower (OR 1.8; 95% CI, 1.4‐2.2) than in trials without a run‐in period (OR 2.0; 95% CI, 1.3‐3.1). Use of placebo or duration of run‐in did not seem to reduce the risk of EPS further.

The data of all but one trial could be used for the analysis of mortality risk. The risk of mortality was 1.6 (95% CI, 0.7‐3.4) and 1.4 (95% CI, 1.0‐2.0) in trials with and without run‐in, respectively. The risk was slightly lower when no placebo was used, but run‐in duration did not seem to affect it.

The sub‐analyses in trials of atypical antipsychotics only yielded similar results for use of run‐in versus no run‐in on all outcomes (Table 2). In trials of conventional antipsychotics, however, efficacy seemed lower and risk of somnolence and EPS higher in trials with run‐in compared to trials without run‐in, but the number of trials without run‐in was 3 at most and confidence intervals were large.

### 3.2 Run‐in and drop‐out

Thirty‐one of 35 studies reported dropout rates for the antipsychotic and placebo groups (Table 3). The number of participants that dropped out was 1519 of 4963 in the antipsychotic groups (30.6%) and 783 of 2747 in the placebo groups (28.5%). The odds ratio of selective dropout was 1.0 (95% CI, 0.9‐1.2) for trials with a run‐in period and 1.0 (95% CI, 0.7‐1.3) for those without.

**Table 3:** Use of run‐in periods and dropout in antipsychotic trials in dementia

	Selective Dropout^a^		Total Dropout	
	OR (95% CI)	N	Beta (95% CI)	N
Conventional and atypical antipsychotics				
No run‐in	1.0 (0.7‐1.3)	5	ref	
With run‐in	1.0 (0.9‐1.2)	26	0.3 (−15.1 to 15.7)	33
washout only	0.9 (0.7‐1.1)	13	ref	
with placebo/drug	1.2 (1.0‐1.4)	13	−4.7 (−14.9 to 5.5)	27
duration ≤ 1 week	0.9 (0.8‐1.1)	14	ref	
duration > 1 week	1.2 (1.0‐1.5)	12	−5.0 (−14.7 to 4.6)	26
Conventional antipsychotics				
No run‐in	1.4 (0.7‐2.8)	2	ref	
With run‐in	1.0 (0.7‐1.3)	9	16.0 (−7.5 to 39.6)	13
Atypical antipsychotics				
No run‐in	0.9 (0.5‐1.7)^b^	4	ref	
With run‐in	1.0 (0.9‐1.2)	19	−7.7 (−25.1 to 9.7)	23

Abbreviation: OR, odds ratio.

a Drug group versus placebo.

b A random effects model was used.

Total dropout in the studies varied between 0% and 81.7%. The use of a run‐in period was not associated with a decreased total dropout (beta 0.3%; 95% CI, −15.1 to 15.7) (Table 3).

### 3.3 Sensitivity analyses

There were not enough trials with and without run‐in periods for the sensitivity‐analysis of atypical antipsychotics without quetiapine.^57^ The results of the analysis without trials that tested both haloperidol and an atypical drug confirmed the pattern of higher efficacy and lower risk of side effects for the conventional and atypical antipsychotic drugs in trials with versus without run‐in(Table S1).

## 4 DISCUSSION

We assessed the association between use of a run‐in period and clinical outcomes of 35 antipsychotic trials in dementia. The reduction in neuropsychiatric symptoms of antipsychotics versus placebo was somewhat higher in trials with a run‐in period than in trials without a run‐in period. The risk of somnolence, EPS, and mortality was lower in trials with than without run‐in. Accordingly, the risk of dropout in the antipsychotic compared with the placebo groups, which represents the balance between beneficial and harmful effects, was not affected when run‐in was used. Use of run‐in periods did not influence total dropout either.

Several reviews have reported an association between the use of run‐in periods and the efficacy of psychotropics. Two meta‐analyses of antidepressant trials showed that a placebo run‐in period was associated with higher effectiveness and more power.^7^, ^8^ We found that the use of run‐in periods was associated with a small increase in efficacy of antipsychotics in dementia. Additionally, we found an association between run‐in periods and a decreased risk of side effects of antipsychotics in dementia. The exclusion of placebo‐responders and drug‐intolerant patients after the run‐in period might have led to increased efficacy and a more favorable side effect profile. The effect of run‐in periods on outcomes of trials has not been investigated often and remains an under‐investigated and likely underestimated source of bias.

A common argument for use of run‐in periods is the reduction of noncompliance and dropout. Our findings showed that a placebo run‐in was not associated with lower between‐group or total dropout rate.

### 4.1 Bias due to run‐in periods

In the studies that we identified, patients that met the inclusion criteria could have been excluded from trial participation as a result of the outcomes during the run‐in period. It is therefore not surprising that the use of run‐in period yielded higher efficacy estimates and lower risks of side effects.^3^, ^58^ In observational studies, bias due to (de)selection of patients based on prior treatment and its outcomes, whether before or after the start of the study, is generally called selection bias.^59^

Bias due to run‐in periods in trials is not commonly discussed in the literature. Most tools for risk of bias assessment in trials do not require consideration of run‐in periods either. In eight of 11 meta‐analyses of antipsychotic trials in dementia, more than 80% of the included trials used a run‐in period (Table S2).^60^, ^61^, ^62^, ^63^, ^64^, ^65^, ^66^, ^67^, ^68^, ^69^, ^70^ Risperidone and olanzapine, currently the two most popular antipsychotic drugs for use in dementia, have been tested in ten trials of which nine included a run‐in period. The general assumption is that selection of patients before randomization is said to reduce only generalizability of study results, not the internal validity of the trial results.^1^

We propose a different view. The screening and selection of patients before randomization should be based on (contra‐)indications that are applied in daily medical practice. The selected patients will then represent the patients of interest to doctors and the population of interest as defined in the PICOs of reviews. This selection needs to be distinguished from deselection of eligible patients based on observed treatment effects during run‐in (between screening and randomization).^2^ The remaining randomized group does not represent the population of interest any more. Estimates of efficacy and risk of side effects will be biased for the population of interest. Therefore, run‐in needs to be considered as a source of bias in trials and reviews of trials.

Another issue to consider is the ethics of entering patients who are doing (relatively) well on a certain antipsychotic drug into a trial of another or the same antipsychotic. During washout, symptoms could return, and it is questionable whether the patient will respond as favorably to a new drug. Especially when it is difficult to convince patients to use antipsychotics and find an antipsychotic that has the desired effect, which is often the case in schizophrenia, switching to another drug for the sake of a trial is even more questionable. Including new instead of prevalent users in trials would be preferable, as is the recognized practice in observational epidemiology.

### 4.2 Strengths and limitations

To our knowledge, this is the first study that investigated the relationship of run‐in periods with clinical outcomes of antipsychotic trials including side effects. Our pooled estimates of efficacy seemed low but are corroborated by previous reviews reporting SMDs between 0.12 and 0.21.^61^, ^66^, ^70^ SMDs above the threshold of 0.200 for a small treatment effect were mainly found in meta‐analyses that focused on aggression or agitation.^63^, ^64^, ^68^, ^70^

A limitation of our research was that only six studies did not use a run‐in phase. Most of these studies were performed in outpatients and with atypical antipsychotics, in particular quetiapine. As a result of this distribution, the higher efficacy in trials with run‐in might be partly attributable to a higher efficacy of conventional antipsychotics. Nevertheless, our sensitivity analysis in atypical antipsychotic trials showed a higher efficacy for trials with a run‐in period as well. Additionally, one would expect the risk of side effects to increase with run‐in as well, but it did not.

## 5 CONCLUSION

The use of a run‐in period is very common in antipsychotic trials for dementia. In these trials, efficacy was higher compared with trials without run‐in, while the risk of side effects was lower. Therefore, the use of a run‐in period in trials might have led to overestimated efficacy and especially underestimated side effects of antipsychotics for neuropsychiatric symptoms in dementia. Meta‐analyses should include sensitivity‐analyses of trials with and without run‐in periods.

## AUTHOR CONTRIBUTIONS

T.A. Hulshof and H.J. Luijendijk searched and selected the trials, extracted the data, and drafted the manuscript. T.A. Hulshof performed the data‐analysis. H.J. Luijendijk designed the study. S.U. Zuidema and C.C. Gispen‐de Wied critically commented on the design and results of the study. All authors reviewed the manuscript and suggested revisions.

## Supporting information


**Table S1.** Use of run‐in periods and the clinical outcomes of antipsychotics in placebo‐controlled trials with conventional OR/AND atypical group
**Table S2**. Trials with run‐in in meta‐analyses of antipsychotics in dementia
