# Supplementary material for: Run‐in periods and clinical outcomes of antipsychotics in dementia: A meta‐epidemiological study of placebo‐controlled trials
Source: Pharmacoepidemiol Drug Saf. 2019 Nov 15;29(2):125–33. doi: 10.1002/pds.4903 (PMC7027584; doi:10.1002/pds.4903)
Supplement: Supplementary file 1 — Table S1. Use of run‐in periods and the clinical outcomes of antipsychotics in placebo‐controlled trials with conventional OR/AND atypical group Table S2. Trials with run‐in in meta‐analyses of antipsychotics in dementia [file PDS-29-125-s001.docx]

**APPENDIX**

**Search string**

**In title/ abstract:** chlorpromazine OR levomepromazine OR promazine OR acepromazine OR triflupromazine OR cyamemazine OR chlorproethazine OR dixyrazine OR fluphenazine OR perphenazine OR prochlorperazine OR thiopropazate OR trifluoperazine OR acetophenazine OR thioproperazine OR butaperazine OR perazine OR periciazine OR thioridazine OR mesoridazine OR pipotiazine OR haloperidol OR trifluperidol OR melperone OR moperone OR pipamperone OR bromperidol OR benperidol OR droperidol OR fluanisone OR oxypertine OR molindone OR sertindole OR ziprasidone OR lurasidone OR flupentixol OR clopenthixol OR chlorprothixene OR tiotixene OR zuclopenthixol OR fluspirilene OR pimozide OR penfluridol OR loxapine OR clozapine OR olanzapine OR quetiapine OR asenapine OR clotiapine OR sulpiride OR sultopride OR tiapride OR remoxipride OR amisulpride OR veralipride OR levosulpiride OR prothipendyl OR risperidone OR mosapramine OR zotepine OR aripiprazole OR paliperidone OR iloperidone OR cariprazine OR brexpiprazole OR pimavanserin

**AND**

**Anywhere:** trial

**AND**

**Anywhere:** dementia

**ETable 1 Use of run-in periods and the clinical outcomes of antipsychotics in placebo-controlled trials with conventional OR/AND atypical group**

|  | **Trials with conventional OR atypical group** | | | | | | | | **Trials with conventional AND atypical group** | | | | | | | |
| --- | --- | --- | --- | --- | --- | --- | --- | --- | --- | --- | --- | --- | --- | --- | --- | --- |
|  | **Efficacy** | | **Somnolence** | | **EPS** | | **Mortality** | | **Efficacy** | | **Somnolence** | | **EPS** | | **Mortality** | |
|  | SMD | N | OR | N | OR | N | OR | N | SMD | N | OR | N | OR | N | OR | N |
| **Conventional antipsychotics** | | | | | | | | | | | | | | | | |
| No run-in | - | 0 | 29.1  (1.5; 578.7) | 1 | 12.4*  (0.6; 246.1) | 1 | 1.0  (0.1; 8.6) | 2 | -0.389  (-0.669; -0.110) | 1 | 1.2  (0.4; 3.1) | 1 | 2.4  (1.2; 4.6) | 1 | 2.1  (0.2; 23.1) | 1 |
| With run-in | -0.424†  (-0.754; -0.095) | 7 | 3.1  (1.3; 7.1) | 2 | 3.2  (1.4; 7.6) | 2 | 0.7  (0.2; 2.3) | 9 | -0.310  (-0.505; -0.115) | 2 | 7.5  (2.5; 23.0) | 2 | 3.0  (1.8; 5.0) | 2 | 1.5  (0.6; 3.6) | 2 |
| **Atypical antipsychotics** | | | | | | | | | | | | | | | | |
| No run-in | -0.056  (-0.208; 0.097) | 3 | 4.9  (2.4; 9.9) | 2 | 2.2  (0.7; 6.6) | 3 | 1.7  (0.7; 4.1) | 3 | -0.387  (-0.665; -0.109) | 1 | 1.0  (0.4; 2.8) | 1 | 0.9  (0.4; 1.8) | 1 | 1.0  (0.1; 16.4) | 1 |
| With run-in | -0.143  (-0.206; -0.081) | 17 | 2.4  (1.9; 3.1) | 12 | 1.7  (1.3; 2.2) | 10 | 1.6  (1.1; 2.4) | 17 | -0.072  (-0.266; 0.122) | 2 | 5.0  (2.4; 10.6) | 2 | 1.1  (0.6; 2.0) | 2 | 0.5  (0.1; 1.5) | 2 |

EPS= Extrapyramidal symptoms; SMD= Standardized mean difference; OR=Odds ratio; † A random effects model was used; $ trials with an atypical drug, a conventional drug and placebo group.

**ETable 2 Trials with run-in in meta-analyses of antipsychotics in dementia**

| **Meta-analysis** | **Included trials, n** | **Trials with run-in, n (%)** |
| --- | --- | --- |
| Farlow 2017^55^ | 16* | 13 (81) |
| Tan 2015^57^ | 10 | 7 (70) |
| Ma 2014^58^ | 16 | 12 (75) |
| Wang 2014^56^ | 6 | 5 (83) |
| Seitz 2013^59^ | 14 | 12 (86) |
| Maher 2011^60^ | 13 | 13 (85) |
| Cheung 2011^61^ | 5 | 2 (40) |
| Lonergan 2010^62^ | 5 | 4 (80) |
| Carson 2006^63^ | 7 | 6 (86) |
| Ballard 2006^64^ | 15 | 12 (80) |
| Lee 2004^65^ | 5 | 5 (100) |

* Individual placebo-controlled start (not extension or stop) trials deduced from references
